# Analysis of bronchoalveolar lavage fluid proteome from systemic sclerosis patients with or without functional, clinical and radiological signs of lung fibrosis

**DOI:** 10.1186/ar2067

**Published:** 2006-10-17

**Authors:** AM Fietta, AM Bardoni, R Salvini, I Passadore, M Morosini, L Cavagna, V Codullo, E Pozzi, F Meloni, C Montecucco

**Affiliations:** 1 Department of Haematological, Pneumological and Cardiovascular Sciences, University of Pavia, Via Taramelli 5, 27100 Pavia, Italy; 2 Department of Biochemistry, University of Pavia, Via Taramelli 5, 27100 Pavia, Italy; 3 Department of Internal Medicine, University of Pavia, Via Taramelli 5, 27100 Pavia, Italy; 4 IRCCS San Matteo, Piazzale Golgi 2, 27100 Pavia, Italy

## Abstract

Lung fibrosis is a major cause of mortality and morbidity in systemic sclerosis (SSc). However, its pathogenesis still needs to be elucidated. We examined whether the alteration of certain proteins in bronchoalveolar lavage fluid (BALF) might have a protective or a causative role in the lung fibrogenesis process. For this purpose we compared the BALF protein profile obtained from nine SSc patients with lung fibrosis (SSc_Fib+_) with that obtained from six SSc patients without pulmonary fibrosis (SSc_Fib-_) by two-dimensional gel electrophoresis (2-DE). Only spots and spot-trains that were consistently expressed in a different way in the two study groups were taken into consideration. In total, 47 spots and spot-trains, corresponding to 30 previously identified proteins in human BALF, showed no significant variation between SSc_Fib+ _patients and SSc_Fib- _patients, whereas 24 spots showed a reproducible significant variation in the two study groups. These latter spots corresponded to 11 proteins or protein fragments, including serum albumin fragments (13 spots), 5 previously recognized proteins (7 spots), and 4 proteins (3 spots) that had not been previously described in human BALF maps, namely calumenin, cytohesin-2, cystatin SN, and mitochondrial DNA topoisomerase 1 (mtDNA TOP1). Mass analysis did not determine one protein-spot. The two study groups revealed a significant difference in BALF protein composition. Whereas levels of glutathione S-transferase P (GSTP), Cu–Zn superoxide dismutase (SOD) and cystatin SN were downregulated in SSc_Fib+ _patients compared with SSc_Fib- _patients, we observed a significant upregulation of α1-acid glycoprotein, haptoglobin-α chain, calgranulin (Cal) B, cytohesin-2, calumenin, and mtDNA TOP1 in SSc_Fib+ _patients. Some of these proteins (GSTP, Cu–Zn SOD, and cystatin SN) seem to be involved in mechanisms that protect lungs against injury or inflammation, whereas others (Cal B, cytohesin-2, and calumenin) seem to be involved in mechanisms that drive lung fibrogenesis. Even if the 2-DE analysis of BALF did not provide an exhaustive identification of all BALF proteins, especially those of low molecular mass, it allows the identification of proteins that might have a role in lung fibrogenesis. Further longitudinal studies on larger cohorts of patients will be necessary to assess their usefulness as predictive markers of disease.

## Introduction

The analysis of the bronchoalveolar lavage fluid (BALF) proteome can potentially provide important information about changes in protein expression and secretion during the course of pulmonary disorders. At the moment, more than 100 human proteins or protein isoforms have been identified in human BALF by using two-dimensional gel electrophoresis (2-DE), including both proteins released locally in the lung by inflammatory or epithelial cells and proteins derived from serum by diffusion across the capillary–alveolar barrier [1,2]. Previous studies showed that the protein composition of BALF was altered in sarcoidosis, idiopathic pulmonary fibrosis, allergic asthma, and chronic obstructive pulmonary disease [3-5]. Furthermore, Rottoli and colleagues [6] recently demonstrated that systemic sclerosis patients with pulmonary fibrosis (SSc_Fib+_) showed a BALF profile that was intermediate between that of patients with sarcoidosis and idiopatic pulmonary fibrosis, despite some particularities.

Systemic sclerosis (SSc) is an autoimmune disorder of unknown origin, characterized by an excessive deposition of collagen and other extracellular matrix proteins on the skin as well as multiple internal organs, alterations in microvasculature, and humoral and cellular abnormalities [7]. Clinically, the disease is very heterogeneous, ranging from limited skin involvement with limited systemic alterations to clinical pictures with diffuse skin sclerosis and severe visceral involvement with fibrosis, microvascular abnormalities and mononuclear cell infiltration of the gastrointestinal tract, lungs, heart, and kidneys. Pulmonary involvement has emerged as potentially the most serious visceral lesion in SSc. Alveolitis is more commonly seen in SSc with diffuse skin sclerosis, particularly in the presence of serum anti-topoisomerase I (anti-Scl70) antibodies, but only 15% of patients eventually reach severe end-stage lung fibrosis [8].

Given the complexity of the pathogenesis of lung fibrosis and the lack of biological markers that can reliably predict which patients will evolve the disease, great interest has been shown in these issues, also comparing cohorts of SSc patients with or without lung involvement. The ability to identify the proteins that are differently expressed in the lower airways of SSc_Fib+ _patients from those in SSc_Fib- _patients might provide new insight into the disease pathogenesis and into the identification of possible protein biomarkers of the disease's progression. With this aim, we used the 2-DE analysis to assess and compare the protein profile of BALFs from a group of SSc_Fib+ _and SSc_Fib- _patients, respectively.

## Materials and methods

### Patients

The samples consisted of archived BALF samples acquired between March 2004 and April 2005 from 15 SSc patients who satisfied the preliminary American College of Rheumatology criteria for disease classification [9]. All the patients were female and non-smoker outpatients at the Rheumatology Unit of the Policlinico San Matteo, Pavia, Italy. They all gave their informed consent to undergo bronchoscopy, and none were being treated with prednisone or other immunosuppressive agents at the time of enrollment. Five patients had SSc with limited skin involvement and 10 had SSc with diffuse skin sclerosis; indirect immunofluorescence on Hep-2 cells detected the antinuclear antibodies, and the anti-Scl70 antibodies were evaluated by enzyme-linked immunosorbent assay (Diamedix Co., Miami, FL, USA). All the patients tested positive for antinuclear antibodies, and nine had anti-Scl70 antibodies.

The diagnosis of SSc-associated lung fibrosis was based on clinical, functional and radiographic signs. In all the cases, carbon monoxide transfer, forced vital capacity, chest radiography, and high-resolution computed tomography (HRCT) determined the respiratory involvement, which was defined as at least a grade 1 severity on the disease severity scale for SSc [10]. In all the cases, the same expert radiologist calculated the Kazerooni score for fibrosis as described previously [11]. Nine patients showed a decrease of at least 20% with regard to the expected carbon monoxide transfer or forced vital capacity values [12]. This decrease was associated with abnormal chest radiograph and the presence of significant fibrosis on the HRCT scan (mean Kazerooni fibrosis score: 10 ± 5 SD). These nine patients were included in the SSc_Fib+ _group. The remaining six patients, who revealed no evidence of lung involvement either on the lung function tests or on the HRCT scan (absence of fibrosis and/or a ground-glass pattern), were included in the SSc_Fib- _group. Table 1 details the patients' demographic, clinical, immunological and functional characteristics.

### Bronchoalveolar lavage and phenotype analysis

Bronchoalveolar lavage (BAL) was performed as described previously [13]. To summarize briefly, the distal tip of the bronchoscope was wedged into the middle lobe or lingular bronchus. A total of 150 ml of warm sterile saline solution were instilled in 30 ml aliquots and serially recovered by gentle aspiration. The first collected aliquot was used for the microscopic and cultural examination of common bacteria and fungi, and of direct acid-fast bacilli smears (Kinyoun method) and cultures for mycobacteria, and for the microscopic examination of *Pneumocystis jiroveci *(Gomori-Grocott silver stain). All the other aliquots were used to assess the total and differential cell counts (performed on cyto-centrifuged preparations with the use of May–Grünwald–Giemsa and Papanicolaou stainings) and the percentages of CD4^+ ^and CD8^+ ^T cells (assessed by cytofluorimetry). Samples were immediately filtered twice through gauze, then centrifuged at 1,500 r.p.m. for 10 min, and BALF samples were promptly frozen in aliquots and stored at -80°C until used.

### Two-dimensional gel electrophoresis

BALF samples were dialyzed (tubing cut-off 1 kDa) for a few days against several changes of distilled water, in the presence of protease inhibitors (protease inhibitor cocktail; Sigma, St Louis, MO, USA), and the protein content was assessed (bicinchoninic acid protein assay kit; Pierce, Rockford, IL, USA). Samples were freeze-dried and pellets were suspended in 8 M urea, 4% w/v CHAPS, 65 mM 1,4-dithioerythritol (solution A). Two-dimensional gel electrophoresis was performed as described previously [14]. In brief, for each analytical experiment the same amount (70 μg) of proteins was loaded on immobilized pH gradient (IPG) strips (pH 3.5 to 10, 18 cm; Amersham Biosciences, Uppsala, Sweden), which were swollen in solution A plus 0.8% carrier ampholytes (Ampholine™, pH 3.5 to 10 for IEF; Amersham Biosciences) and a trace of bromophenol blue. A larger amount of protein (1 mg) had to be loaded for identification by mass spectrometry (MS). Rehydration was performed in the strip holder of the IPGphor system at 20°C (Amersham Biosciences) and isoelectrofocusing was terminated at 60 kV h. The IPG strips were equilibrated first for 12 minutes in urea/SDS/Tris buffer, and then in the same buffer containing 2.5% (w/v) iodoacetamide. The run in the second dimension was performed on 9 to 16.5% polyacrylamide linear gradient gels with a constant current of 40 mA per gel at 10°C. The gels were stained with ammoniacal silver nitrate or colloidal Coomassie G-250 [15].

### Evaluation and protein identification

The Versadoc Imaging System Model 3000 (Bio-Rad, Richmond, CA, USA) scanned the gels, which the PD QUEST 7.1 software (Bio-Rad) then analyzed. The total number of spots, the non-matched spots and the spot parameters were measured. To detect differentially expressed protein spots in SSc_Fib+ _compared with those in SSc_Fib- _patients, the software was instructed to create one reference map for each patient group. The software established these maps by taking into account only the spots that were constantly expressed in each patient of the same group. At least two gels from each patient were evaluated. Initially, we compared the two reference maps so as to assess differences in spot quantity between the two study groups. The PD QUEST 7.1 software determined the spot quantity by formula: Spot height × π × σ_*x *_× σ_*y *_(where the spot height represented the peak of the spot's Gaussian representation, and σ_*x *_and σ_*y *_were the SD of the spot's Gaussian distribution in the directions of the *x *and *y *axes, respectively) normalized the value for the total protein content of each gel. The differences between the two study groups were then confirmed by analysing the spot quantities observed in single patients. On the basis of the recent guidelines for proteomics research [16], non-matched spots and spots that differed significantly in quantity (*p *< 0.05) were regarded as differentially expressed in SSc_Fib+ _compared with SSc_Fib- _patients.

Proteins were identified by comparing the BALF maps obtained in this study with the SWISS-2D PAGE human plasma map [17] and published BALF maps [5,6], using immunoblotting, matrix-assisted laser desorption/ionization MS, and/or liquid chromatography MS/MS. For the MS analysis, the selected spots, excised from Coomassie-stained gels, were analyzed in a Voyager DE-PRO mass spectrometer (Applied Biosystems, Foster City, CA, USA), equipped with a reflectron analyzer and used in delayed extraction mode. Mass signals were used for database searching with the MASCOT peptide fingerprinting search program (Matrix Science, Boston, MA, USA). A quadrupole time-of-flight. Ultima hybrid mass spectrometer (Micromass, Toronto, Ontario, Canada) was used for the liquid chromatography–MS/MS analysis. The instrument was set up in a data-dependent MS/MS mode, and the raw MS and MS/MS spectra were elaborated with ProteinLynx software. The MASCOT MS/MS ion search software for protein identification was used. These evaluations were performed at CEINGE Biotecnologie Avanzate s.c. a r.l., University Federico II, Naples, Italy).

### Statistical methods

Results are presented as mean ± SD or median and interquartile range. Normally distributed variables were analyzed by a two-tailed Student's *t *test. Proteomics expression data were compared by using the Mann–Whitney *U *test. For all tests *p *< 0.05 was regarded as significant. Analyses were performed with PRISM 3.0 (GraphPad Software, San Diego, CA, USA).

## Results

BAL was performed in 15 SSc patients, including 9 patients with clinical, functional and HRCT signs of interstitial lung fibrosis (SSc_Fib+ _patients) and 6 patients who had no signs and symptoms of lung involvement (SSc_Fib- _patients). No clinical or microbiological evidence of bacterial or fungal infections was ever found in the included patients. Furthermore, the two patient groups revealed no significant difference in total BAL cells recovered, CD4^+^/CD8^+ ^T-cell ratio, protein content, or differential BAL cell counts, although the SSc_Fib+ _patients displayed a trend toward higher neutrophil and eosinophil counts (Table 2). Neutrophil and eosinophil counts, as expected, were significantly higher in the SSc_Fib+ _patients than in data obtained in our laboratory from a group of healthy volunteers [12].

### BALF proteome in SSc_Fib+ _and SSc_Fib- _patients

**Figure 1:**
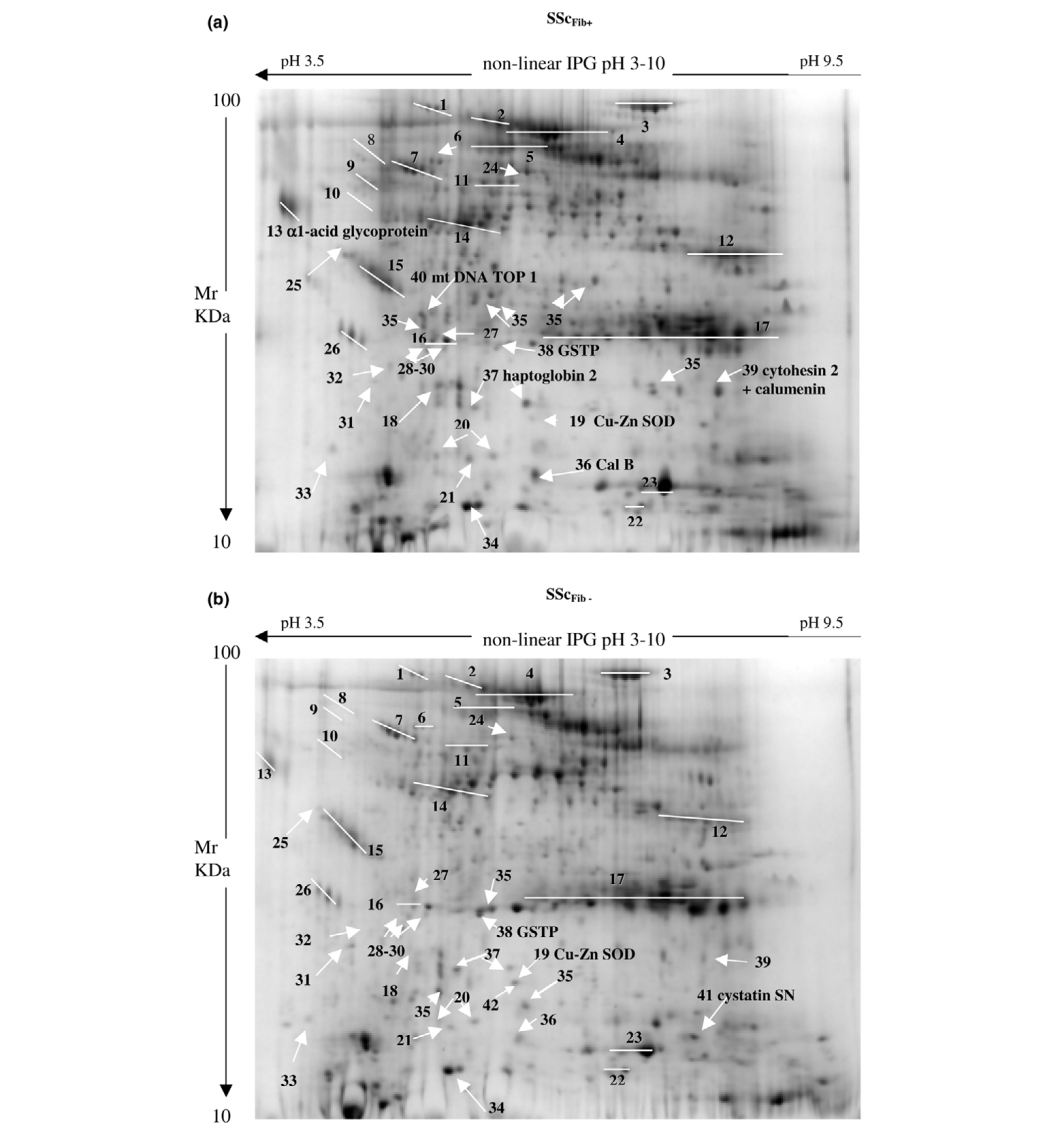
Representative Coomassie-stained gels of bronchoalveolar lavage fluid. Samples shown were taken from a systemic sclerosis patient with lung fibrosis (SSc_Fib+_) **(a) **and a systemic sclerosis patient without lung fibrosis (SSc_Fib-_) **(b)**. Analyzed spots are marked with a number and the corresponding identified proteins are listed in Tables 3 and 4. Proteins whose expression was significantly different between SSc_Fib+ _patients and SSc_Fib- _patients are also indicated by name.

Two-dimensional gel electrophoresis analyzed the whole protein profile of BALF from SSc_Fib+ _and SSc_Fib- _patients, to evaluate possible differences in protein expression between the two states of the disease. The mean number of spots, of which several were organized as spot-trains, in gels of BALF from SSc_Fib+ _and SSc_Fib- _patients was 365 ± 84 and 390 ± 75, respectively. However, only 332 and 348 of these spots were constantly expressed in BALF from all single SSc_Fib+ _and SSc_Fib- _patients, respectively. Figure 1 shows a representative Coomassie-stained gel map from an SSc_Fib+ _patient (Figure 1a) and an SSc_Fib- _patient (Figure 1b). By comparing the quantity of 59 individual spots and 12 spot-trains, we identified 35 spots and 12 spot-trains whose quantity did not vary significantly between SSc_Fib+ _and SSc_Fib- _patients (Mann–Whitney *U *test *p *> 0.05). By matching these gel maps with published two-dimensional BALF maps, we identified 30 proteins corresponding to the 47 spots and spot-trains that did not vary in quantity between the two patient groups (Table 3). Immunoblotting also validated the assignment of some of these proteins. Furthermore, we detected 24 individual spots that showed a reproducible, significant variation between the two study groups. The quantity in 21 of these spots was significantly increased, whereas the quantity in 3 spots was significantly decreased in SSc_Fib+ _patients compared with SSc_Fib- _patients.

Matrix-assisted laser desorption/ionization MS with or without liquid chromatography–MS/MS analysis identified the proteins corresponding to 21 spots that were constantly and significantly expressed in a different way between the two study groups. A comparison with published two-dimensional BALF maps followed by immunoblotting validation identified the proteins corresponding to the remaining three spots with the aforementioned criteria (Table 4). These 24 differently expressed spots corresponded to 11 proteins/protein fragments in total: 13 spots corresponded to serum albumin fragments, 7 of them corresponded to 5 previously recognized BALF proteins (α1-acid glycoprotein (2 spots), haptoglobin-α chain (2 spots), calgranulin (Cal) B, Cu–Zn superoxide dismutase (SOD) and glutathione S-transferase P (GSTP)), and 3 spots corresponded to 4 proteins that, to our knowledge, had not previously been described in published BALF two-dimensional maps (calumenin and cytohesin-2 (1 spot), cystatin SN and a fragment of mtDNA TOP1). One protein spot was marked as not determined because its identification gave a score that indicated uncertain identification. SSc_Fib+ _patients showed a significant upregulation of α1-acid glycoprotein, haptoglobin-α chain, Cal B, calumenin, and cytohesin-2 levels compared with SSc_Fib- _patients. Furthermore, the expression of a fragment of mtDNA TOP1 was exclusively detectable in SSc_Fib+ _patients, including two patients who were negative for anti-Scl70 serum antibodies. Finally, whereas Cu–Zn SOD was significantly downregulated, GSTP and cystatin SN were not detectable in SSc_Fib+ _patients in contrast with SSc_Fib- _patients.

## Discussion

The pathogenesis of SSc is extremely complex, and no single unifying hypothesis or pathogenetic mechanism can explain all the aspects of this disease. It is therefore widely accepted that functional alterations of several cell effectors, including fibroblasts, endothelial cells, and T and B lymphocytes, are intimately involved in the development of the clinical and pathological manifestations of SSc [7,18,19]. Abnormalities in lung function occur in about 70% of SSc patients and currently represent the main cause of mortality in this population [7]. However, the factors that drive the severe and progressive visceral fibrosis remain mostly undetermined. Unlike the recent pathogenetic hypothesis for idiopathic interstitial lung fibrosis, which assigns only a marginal role to inflammation, fibrogenesis in SSc-related lung involvement is generally thought to be the result of a complex series of interstitial immunoinflammatory reactions [20]. The persistent and unregulated activation of genes that encode various collagen and other extracellular matrix proteins is regarded as a crucial pathogenetic event [21,22]. Furthermore, an alteration in the production of pro-inflammatory and/or pro-fibrotic cytokines and chemokines, growth factors (in particular transforming growth factor-β), and signaling molecules also seems to have a relevant role in fibrogenesis [23,24]. The identification of proteins that are differently expressed in the airways of SSc_Fib+ _patients compared with patients with no signs or symptoms of lung involvement may cast light on the pathogenetic mechanism of the disease and may thereby indicate protein biomarkers that are useful for the diagnosis of lung involvement, for the monitoring of SSc progression, and for the identification of possible new therapeutic targets. In this context, the use of 2-DE and the mapping of BALF seem highly interesting.

Our results demonstrated that quantitative and qualitative differences can be found between patients with SSc-associated lung fibrosis and patients with SSc with no signs or symptoms of lung involvement. At least 24 well-defined spots showed significant and reproducible variations in relative abundance between the two patient groups. Some of these spots corresponded to previously described BALF proteins and protein fragments, namely α1-acid glycoprotein, GSTP, Cu–Zn SOD, haptoglobin-α chain, and calgranulin B, whereas 4 of the proteins found had not previously been described in published two-dimensional BALF maps, namely cytohesin-2, calumenin, cystatin SN, and human mtDNA TOP1 fragment. In SSc_Fib+ _patients, GSTP and cystatin SN were below the detection limit, whereas Cu–Zn SOD was downregulated. In contrast, α1-acid glycoprotein, calumenin, cytohesin-2, haptoglobin-α chain, and calgranulin B were significantly upregulated, and the expression of mtDNA TOP1 was observed only in SSc_Fib+ _patients.

On the basis of these preliminary results, we can infer that 2-DE analysis of BALF can provide useful insight into the understanding of mechanisms involved in lung fibrogenesis through the identification of proteins that are deregulated in SSc-associated lung fibrosis. However, our results, as well as those of previous studies, clearly indicated that 2-DE analysis did not provide an exhaustive identification of all proteins that can be present in BALF, especially those of low molecular mass such as cytokines, chemokines, growth factors, and signaling molecules. Because these molecules are thought to be relevant to the pathogenesis of SSc-associated lung fibrosis, a combination of 2-DE analysis and proteomic approaches that specifically address the identification of these factors is necessary for the assessment of the whole protein pattern of BALF. Despite these considerations, most of the proteins we found differently regulated between SSc_Fib+ _patients and SSc_Fib- _patients could have a role in the development of lung fibrosis, whereby some of them are involved in the mechanisms that protect against lung injury or inflammation whereas others are involved in the mechanisms that drive fibroproliferation.

Oxidative stress is believed to be important in the pathogenesis of lung damage and in the development of lung fibrosis [25]. Among various enzymatic and non-enzymatic mechanisms that protect cells and tissues from oxidants [26,27], glutathione transferases [28] and SODs [29] are among such mechanisms that are thought to have a key protective role, especially in the lung. High levels of extracellular SODs are thought to have a protective role for lung matrix [29]. Recently, Tourkina and colleagues [30] demonstrated a deficiency in GSTP1 in scleroderma lung fibroblasts. The observation that GSTP and Cu–Zn SOD levels are severely downregulated in BALF from SSc_Fib+ _patients is in line with these findings, and confirms the relevance of these molecules as a defense tool for the lung against oxidant-induced damage and/or fibroproliferation.

Moreover, in BALF from SSc_Fib+ _patients, we did not detect levels of cystatin SN, which was, in contrast, well represented in SSc_Fib- _patients. Cystatin SN is a secreted protein, which belongs to SD-type or type-2 cystatins [31], which are potent inhibitors of CA clan mammalian cysteine peptidases. Intracellularly, these enzymes participate in normal protein turnover, in antigen and protein processing, and in apoptosis, whereas extracellularly they are involved in the inhibition of the proteolytic cascade [32]. The direct inhibition of endogenous and exogenous cysteine peptidases is the only function described so far for cystatin SN, suggesting that its function is to protect tissues against injury induced by these enzymes. However, recent results obtained with other type-2 cystatins seem to suggest a broader spectrum of activities, including the upregulation of IL-6 by human gingival fibroblasts, interferon-γ expression in CD4^+ ^T cells [33,34], and the involvement in the differentiation and maturation of human dendritic cells [35].

Furthermore, we found an upregulation of at least six proteins in BALF from SSc_Fib+ _patients: haptoglobin-α chain, α1-acid glycoprotein, calumenin, cytohesin-2, Cal B, and mtDNA TOP1.

Haptoglobin and α1-acid glycoprotein are positive acute-phase proteins, whose plasma concentrations increase during inflammation [36]. The increased levels of both proteins in BALF from SSc_Fib+ _patients is likely to originate from the passive diffusion from plasma, as a result of the increased permeability of the air–blood barrier during the course of fibrosing alveolitis.

Calumenin is a member of the CREC family [37] that is secreted into the medium of cultured cells including fibroblasts [38] and early melanosomes [39]. Intracellularly, calumenin has a chaperone function in several Ca^2+^-regulated processes, but extracellularly its function is not yet fully understood. Vorum and colleagues found that calumenin binds to the serum amyloid P component, suggesting that it is involved in the formation of amyloid deposits in different tissues [40]. Coppinger and colleagues recently described the presence of calumenin in platelets and its release on platelet activation, as well as the protein's presence in human atherosclerotic lesions [41], therefore suggesting a possible role of this protein in leading to vascular injury through the promotion of platelet adhesion, fibrinogenesis, and intravascular thrombus deposition. Taking into account that a crucial pathogenetic step in SSc involves injury to microvasculature [19,20], it is possible to infer that calumenin could have a role in taking such an injury to lung level.

Cytohesin-2 is a member of a family of cytoplasmic signaling proteins [42], which may move from the cytosol to the plasma membrane after cell stimulation [43]. Although its biological function is mostly unknown, a recent study demonstrated that cytohesin-2 is an activator of the mitogen-activated protein (MAP) kinase signaling pathway [44]. Because MAP kinases are activator signals for lung fibroblasts [23], we suggest that cytohesin-2 might participate in the development and/or progression of lung fibrosis in SSc patients through the activation of the MAP kinase signaling pathways in lung fibroblasts. This hypothesis needs to be verified with further experimental observations.

Calgranulin B is an S100 protein that is unique together with Cal A in its myeloid-specific expression profile and in its abundance in neutrophils [45]. Increased levels of Cal B are present in the bronchial secretion of patients with chronic inflammatory disease, and these levels may induce the production of IL-8 by airway epithelial cells [46]. Furthermore, Viemann and colleagues demonstrated that the expression of Cal B and Cal A correlates with the inflammatory activity in systemic vasculitis, thereby confirming the role of these proteins in inflammatory reactions of endothelia [47]. Although the general consensus is that the Cal B function depends mainly on heterodimer formation (Cal B–Cal A), several studies have demonstrated that the monomer is also a potent stimulator of neutrophils and is involved in the recruitment of leukocytes to the site of inflammation through the regulation of adhesion and the extravasion of neutrophils [45,48]. Furthermore, recombinant Cal B and its homodimer stimulate the proliferation of rat fibroblasts, suggesting that this protein might also function as a mitogen for fibroblasts in chronic inflammation [49]. On the basis of these observations, Cal B could have a role in SSc-associated lung fibrosis through several pathways, including amplifying the inflammatory process by inducing the extravasion of neutrophils and the production of IL-8, inducing an inflammatory reaction in the endothelia, and/or stimulating lung fibroblast proliferation.

Finally, we found that a fragment of mtDNA TOP1 was detectable only in BALF from patients with interstitial lung fibrosis, including in two SSc_Fib+ _patients who were negative for serum anti-Scl70 antibodies. This protein fragment was never observed in BALF from SSc_Fib- _patients, including in two patients with positive anti-Scl70 antibodies. A common 13-exon motif and a similar amino-acid sequence characterize genes for mitochondrial and nuclear DNA TOP1, except for the N-terminal domain, which in mtDNA TOP1 contains a mitochondrial localization sequence instead of the nuclear localization signal [50]. Although we demonstrated that the presence of detectable levels of mtDNA TOP1 was a constant and particular feature of SSc_Fib+ _patients, we did not fully ascertain the antigenic role of this protein fragment and its relation with the development of lung fibrosis. Because autoantibodies against DNA TOP1 are strongly correlated with interstitial pulmonary involvement in SSc [51], further studies are warranted to assess the role and the frequency of this antigenic target in BALF.

## Conclusion

**Figure 2:**
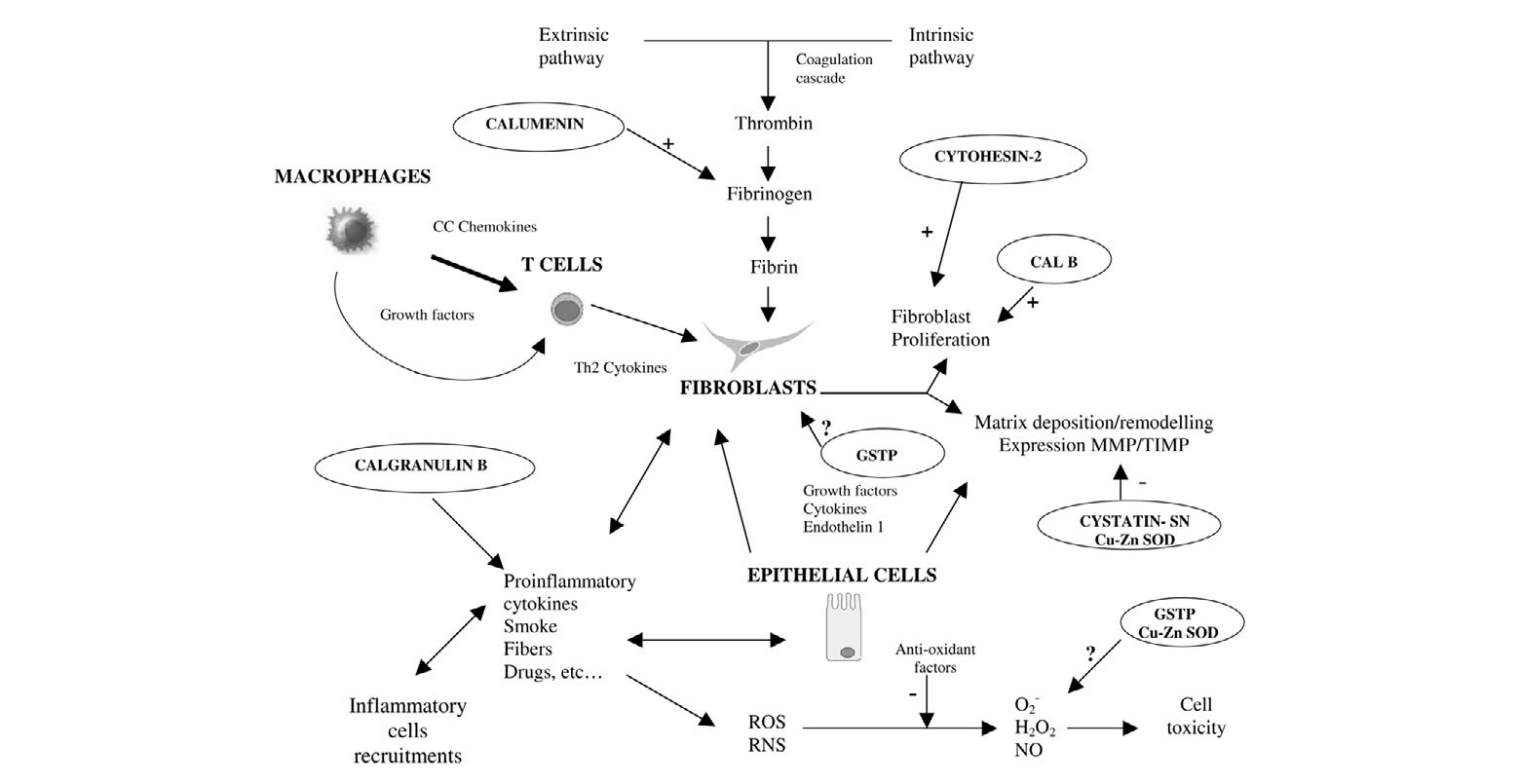
Hypothetical role of some disregulated BALf proteins in mechanisms driving SSc-related lung fibrosis. Inflammation is thought to be the main mechanism driving lung fibrosis in scleroderma patients. In this complex inflammatory process several pathways are involved, including the activation of T cells and epithelial cells, the secretion of pro-inflammatory cytokines and growth factors, and fibroblast proliferation. Furthermore, products from the coagulation cascade and oxidative stress may contribute to fibrogenesis. The upregulation of calgranulin B (Cal B), cytohesin-2 and calumenin might favor inflammation and fibrogenesis, whereas the downmodulation of the protective factors glutathione S-transferase P (GSTP), Cu–Zn superoxide dismutase (SOD) and cystatin SN may amplify tissue injury and inflammation. MMP, matrix metalloproteinases; TIMP, tissue inhibitor of matrix metalloproteinases; ROS, reactive oxygen species; RNS, reactive nitrogen species; O_2_^-^, superoxide; H_2_O_2_, hydrogen peroxide; NO, nitric oxide; SSc_Fib+_, systemic sclerosis patients with lung fibrosis; SSc_Fib-_, systemic sclerosis patients without lung fibrosis.

On the basis of these preliminary results we believe that the comparative analysis of BALF proteome from SSc_Fib+ _and SSc_Fib- _patients by 2-DE might provide a useful insight into some relevant steps of lung fibrogenesis. SSc-associated lung fibrosis is a multifaceted process involving the activation of T cells and epithelial cells, the secretion of pro-inflammatory cytokines and growth factors, and fibroproliferation (Figure 2). In addition, several factors, including activation of the coagulation cascade and oxidative stress, are thought to influence the development of lung fibrosis. At least six proteins that were differently regulated in BALF from SSc_Fib+ _patients and in BALF from SSc_Fib- _patients might have a role in the pathogenesis of SSc-associated interstitial lung disease. As shown in Figure 2, the upregulation of Cal B, cytohesin-2, and calumenin in BALF from SSc_Fib+ _patients could further promote the synthesis of pro-inflammatory cytokines, the influx of inflammatory cells, and fibrinogenesis, whereas the downregulation of GSTP, Cu–Zn SOD and cystatin SN could favor vascular and lung tissue injury through the impairment of mechanisms that protect against oxidants and cysteine peptidases. However, further animal and human studies will be necessary both to assess the actual role of each of these factors in the development of SSc-associated lung fibrosis and to determine whether these factors are relevant as disease biomarkers, and if so which ones. Conclusions from these studies could enable us to predict which patients will develop progressive pulmonary fibrosis.

## Abbreviations

Anti-Scl70 = anti-topoisomerase I antibodies; BAL = bronchoalveolar lavage; BALF = bronchoalveolar lavage fluid; Cal = calgranulin; 2-DE = two-dimensional gel electrophoresis; GSTP = glutathione S-transferase P; HRCT = high-resolution computed tomography; IL = interleukin; IPG = immobilized pH gradient; MAP = mitogen-activated protein; MS, mass spectrometry; mtDNA TOP1 = mitochondrial DNA topoisomerase 1; SOD = superoxide dismutase; SSc = systemic sclerosis; SSc_Fib- _= systemic sclerosis patients without lung fibrosis; SSc_Fib+ _= systemic sclerosis patients with lung fibrosis; TLCO = carbon monoxide transfer.

## Competing interests

The authors declare that they have no competing interests.

## Authors' contributions

FAM generated and planned the project, supervised the work and wrote the manuscript. BAM and SR contributed equally to perform the research, analyzed critically data and participated in the preparation of the manuscript. PI assisted in designing the experiments and conducted 2-DE and immunoblotting. MM was involved in data analysis and performed statistical analysis. CL and CV selected patients and revised all clinical charts. PE was involved in the coordination of the study and in the discussion and revision of the manuscript. MF participated in the study design, supervised the work, read the bronchoalveolar lavage and helped to draft the manuscript. MC participated in the study design and its coordination and participated in preparation of the manuscript. All authors read and approved the final manuscript.

## Figures and Tables

**Table 1:** Demographic, clinical, immunological and functional characteristics of scleroderma patients

Characteristic	SSc_Fib+_	SSc_Fib-_
Number	9	6
Age, years (mean ± SD)	55.6 ± 10.3	50.5 ± 9.6
Cutaneous involvement		
Lc SSc (percentage)	1 (11.1)	4 (66.7)
Dc SSc (percentage)	8 (89.9)	2 (33.3)
Skin score (mean ± SD)	17.0 ± 8.0	10.0 ± 6.0
Autoantibodies		
ANA positive (percentage)	9 (100)	6 (100)
Anti-Scl70 positive (percentage)	7 (77.7)	2 (33)
Lung function test		
FVC, percentage predicted (mean ± SD)	80.9 ± 11.7^a^	118.7 ± 11.6
TLCO, percentage predicted (mean ± SD)	60.01 ± 12.6^a^	72.03 ± 13.8
Kazerooni score	10.0 ± 5	0

Skin score and Kazerooni score for fibrosis were determined as described previously [9,11]. SSc_Fib+_, systemic sclerosis patients with functional lung fibrosis and signs of lung fibrosis on high-resolution computed tomography; SSc_Fib-_, SSc patients with no signs and symptoms of lung involvement; Lc SSc, limited cutaneous SSc; Dc SSc, diffuse cutaneous SSc; ANA, antinuclear antibodies; anti-Scl70, anti-topoisomerase I antibodies; FVC, forced vital capacity; TLCO, carbon monoxide transfer. ^a^Decrease more than 20% with regard to the expected TLCO or FVC values.

**Table 2:** Cytology, CD4^+^/CD8^+ ^T-cell ratio and protein contents of bronchoalveolar lavage samples

Parameter	SSc_Fib+ _(*n *= 9)	SSc_Fib- _(*n *= 6)	Controls (*n *= 9)
Total BAL cells (10^5^/ml)	2.05 ± 1.13	1.80 ± 0.74	2.02 ± 1.33
Macrophages (percentage)	84.97 ± 8.66	87.73 ± 5.12	85.78 ± 7.09
Lymphocytes (percentage)	8.01 ± 4.15	7.80 ± 2.63	12.33 ± 6.36
Neutrophils (percentage)	4.44 ± 4.03^a^	2.76 ± 1.98	1.56 ± 1.59
Eosinophils (percentage)	2.62 ± 3.66^a^	0.83 ± 0.60	0.33 ± 0.70
CD4^+^/CD8^+ ^ratio	2.30 ± 1.02	2.14 ± 1.16	2.4 ± 0.71
Protein concentration (μg/ml)	130.44 ± 53.76	133.43 ± 32.38	ND

Results are given as mean ± SD. SSc_Fib+_, systemic sclerosis patients with functional lung fibrosis and signs of lung fibrosis on high-resolution computed tomography; SSc_Fib-_, SSc patients with no signs and symptoms of lung fibrosis. Control values are taken from data previously assessed in a group of healthy volunteers [12]. BAL, bronchoalveolar lavage; ND, not determined. ^a^*p *< 0.05 compared with controls (all assessed by Student's *t *test).

**Table 3:** Proteins whose quantity in BALF did not significantly vary between SSc_Fib+ _and SSc_Fib- _patients

Spot no.	Protein	Identification method
1	1β glycoprotein	GM
2	Hemopexin	GM
3	Transferrin	GM
4	Albumin	GM
5	Ig heavy chain α	GM
6	Vitamin D-binding protein	GM
7	α1-antitrypsin	GM, Ab
8	α1-antichymotrypsin	GM, Ab
9	α1-HS-glycoprotein	GM
10	Leucin-rich α2-glycoprotein	GM
11	Fibrinogen γ A chain	GM, Ab
12	Ig heavy chain γ	GM
14	Haptoglobulin 1β chain	GM
15	Pulmonary surfactant-associated protein A	GM
16	Apolipoprotein A1	GM, Ab
17	Ig light chain κ, λ	GM, Ab
18	Serum retinol-binding protein	GM
20	Transthyretin	GM, Ab
21	Immunoglobulin-binding factor	GM
22	Calgranulin A	GM
23	Hemoglobin β chain	GM
24	ERP60 (protein disulfide isomerase)	GM, Ab
25	α1-antitrypsin (fragment)	GM, Ab
26	Ig J chain	GM
27	C-reactive protein	GM
28–30	Apolipoprotein A1 (fragment)	GM, Ab
31	Calcyphosine	GM
32	TCTP (translationally controlled tumor protein)	GM
33	ATP synthase δ chain mitochondrial	GM
34	L-FABP (liver fatty acid-binding protein)	GM

Spot quantity was assessed by PDQUEST 7.1 software on two BALF gel maps of individual SSc_Fib+ _(*n *= 9) and SSc_Fib- _(*n *= 6) patients. Spot quantity between SSc_Fib+ _and SSc_Fib- _patients did not differ significantly (Mann–Whitney *U *test, *p *> 0.05). Spot no. refers to the annotations in Figure 1. BALF, bronchoalveolar lavage fluid; SSc_Fib+_, systemic sclerosis patients with functional lung fibrosis and signs of lung fibrosis on high-resolution computed tomography; SSc_Fib-_, SSc patients with no signs and symptoms of lung fibrosis; GM, gel matching with plasma two-dimensional gel electrophoresis database from Swiss-PROT [17] and published two-dimensional gel electrophoresis BALF maps [5,6]; Ab, immunoblotting.

**Table 4:** BALF proteins whose regulation in SSc_Fib+ _and SSc_Fib- _patients is different

Spot no.	Median (IQR) spot quantity			Protein or fragment	MW (kDa)	pI	AC	Identification method
						
	SSc_Fib+_	SSc_Fib-_	*p*					
13	328.0 (256.5–482.5)	86.0 (68.6–110.4)	0.0003	α1-acid glycoprotein (orosomucoid)	23.5	4.93	P02763	GM (plasma, BALF), Ab
19	7.9 (5.6–9.6)	28.2 (24.5–32.6)		Cu–Zn superoxide dismutase	15.8	5.70	P00441	GM (plasma, BALF), Ab
35	343.0 (328.5–664.0)	168.0 (131.5–267.0)	0.0041	Serum albumin^a^	69.4	5.92	P02768	MS
36	96.6 (68.9–127.6)	24.4 (17.2–64.5)	0.0012	Calgranulin B (S100A9; MRP14)	13.2	5.71	P06702	MS, GM (plasma, BALF)
37	53.3 (47.4–72.1)	11.1 (8.0–22.9)	0.0001	Haptoglobin (Hp2) α chain^a^	15.9	5.57	P00738	MS, LC, GM (plasma, BALF)
38	Not expressed	32.0 (29.5–39.0)		Glutathione S transferase P	23.2	5.44	P09211	MS, GM (plasma, BALF)
39	59.1 (50.8–73.1)	14.8 (9.4–19.6)	0.0014	Cytohesin-2^a,b^	46.5	5.43	Q99418	MS, LC MS, LC
				Calumenin^a,b^	37.1	4.47	O43852	MS, LC MS, LC
40	29.2 (25.5–30.8)	Not expressed		Mitochondrial DNA topoisomerase 1^a,b^	69.8	9.46	Q969P6	MS, LC
41	Not expressed	45.5 (32.2–58.9)		Cystatin SN^b^	16.3	6.82	P01037	MS, LC
42	Not expressed	37.7 (34.0–41.0)		Not determined	-	-	-	(MS, LC)

Spot quantity values were measured on two BALF gel maps of individual SSc_Fib+ _(*n *= 9) and SSc_Fib- _(*n *= 6) patients by PDQUEST 7.1 software. Results are expressed as median and interquartile range (IQR). Spot no. refers to the annotations in Figure 1; *p *values were calculated with the Mann–Whitney *U *test. BALF, bronchoalveolar lavage fluid; SSc_Fib+_, systemic sclerosis patients with functional lung fibrosis and signs of lung fibrosis on high-resolution computed tomography; SSc_Fib-_, SSc patients with no signs and symptoms of lung fibrosis; MW, theoretical average molecular mass; pI, isoelectric point; AC, accession number from the Swiss-PROT 2DPAGE database [17]; GM, gel matching with plasma two-dimensional gel electrophoresis database from Swiss-PROT [17] and published BALF two-dimensional gel electrophoresis maps [5,6]; Ab, immunoblotting; MS, matrix-assisted laser desorption/ionization mass spectrometry analysis; LC, liquid chromatography–tandem mass spectrometry analysis. ^a^Fragments. ^b^Proteins not previously described in published BALF two-dimensional gel electrophoresis maps.
